# Supplementary material for: Defining the networks that connect RNase III and RNase J-mediated regulation of primary and specialized metabolism in Streptomyces venezuelae
Source: J Bacteriol. 2025 Apr 14;207(5):e00024-25. doi: 10.1128/jb.00024-25 (PMC12096830; doi:10.1128/jb.00024-25)
Supplement: Supplemental figures — Fig. S1 to S5. [file jb.00024-25-s0001.pdf]

Supplementary figures

Figure S1

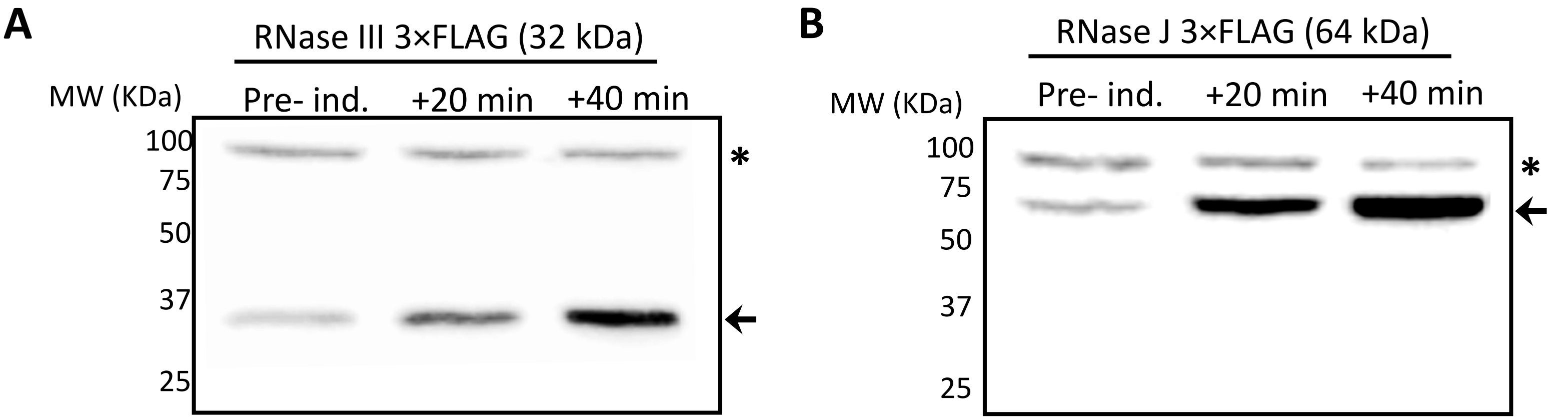

Supplementary Figure 1: RNase III and RNase J are effectively induced following addition of thiostrepton. Immunoblot for **(A)** RNase III 3×FLAG and **(B)** RNase J 3×FLAG using anti-FLAG-specific antibodies, in samples isolated pre-induction and 20- and 40-minutes post-induction. Arrow heads indicate the induced RNase III (left) and RNase J (right). Asterisks mark a non-specific band that served as a protein loading control.

Figure S2

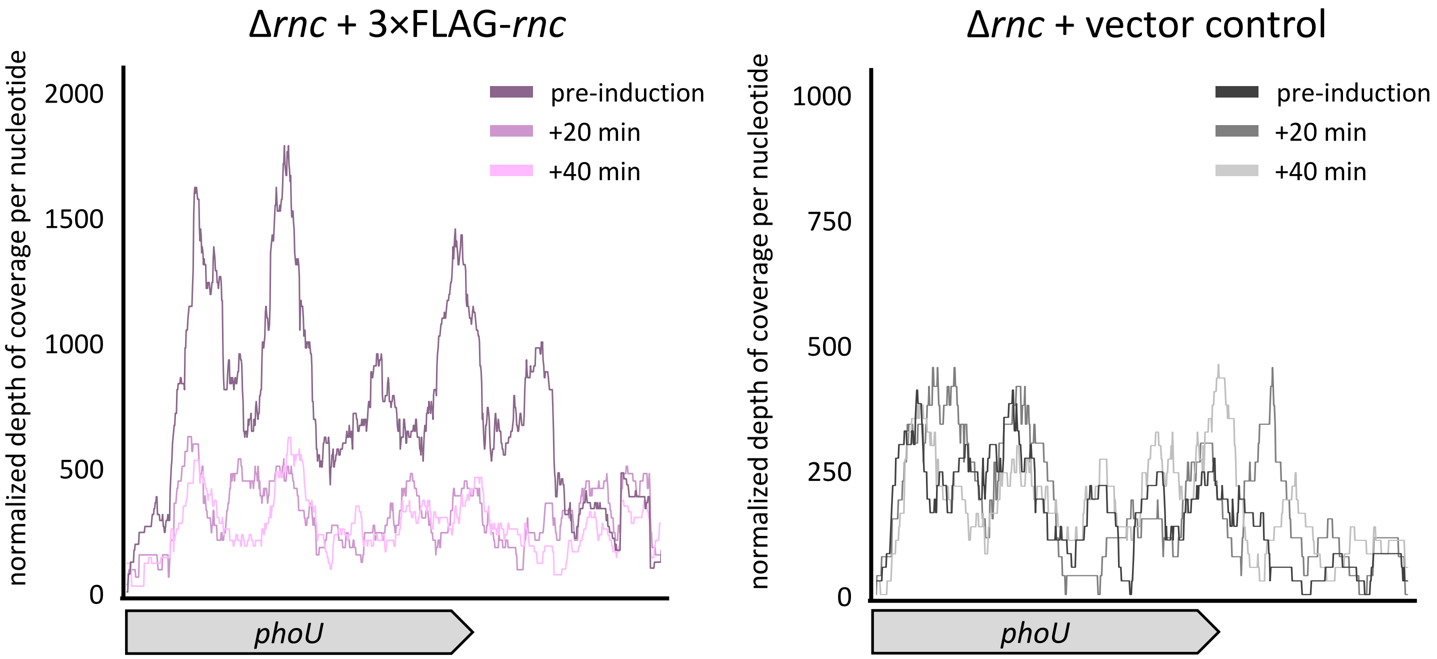

Supplementary Figure 2: *phoU* transcript is directly targeted by RNase III. Second biological replicate for experiment presented in Figure 5C.

Figure S3

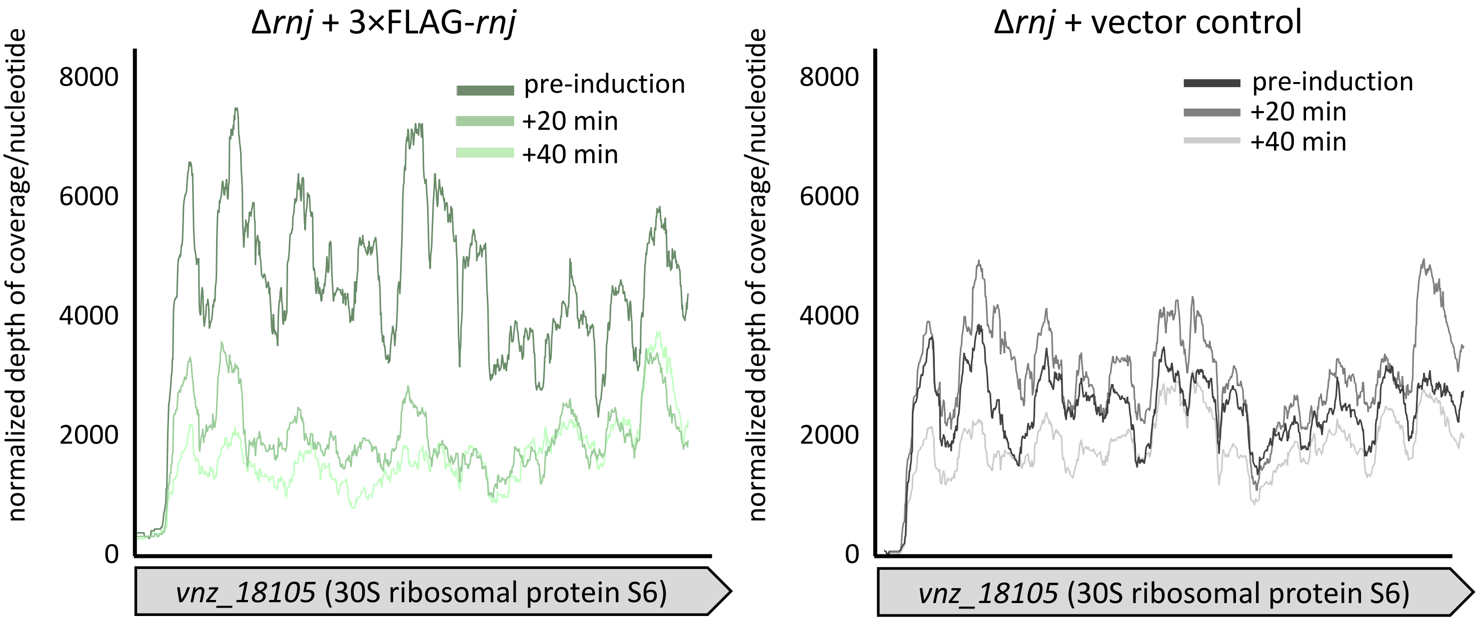

Supplementary Figure 3: *vnz_18105* transcript is directly targeted by RNase J. Second biological replicate for experiment presented in Figure 6.

Figure S4

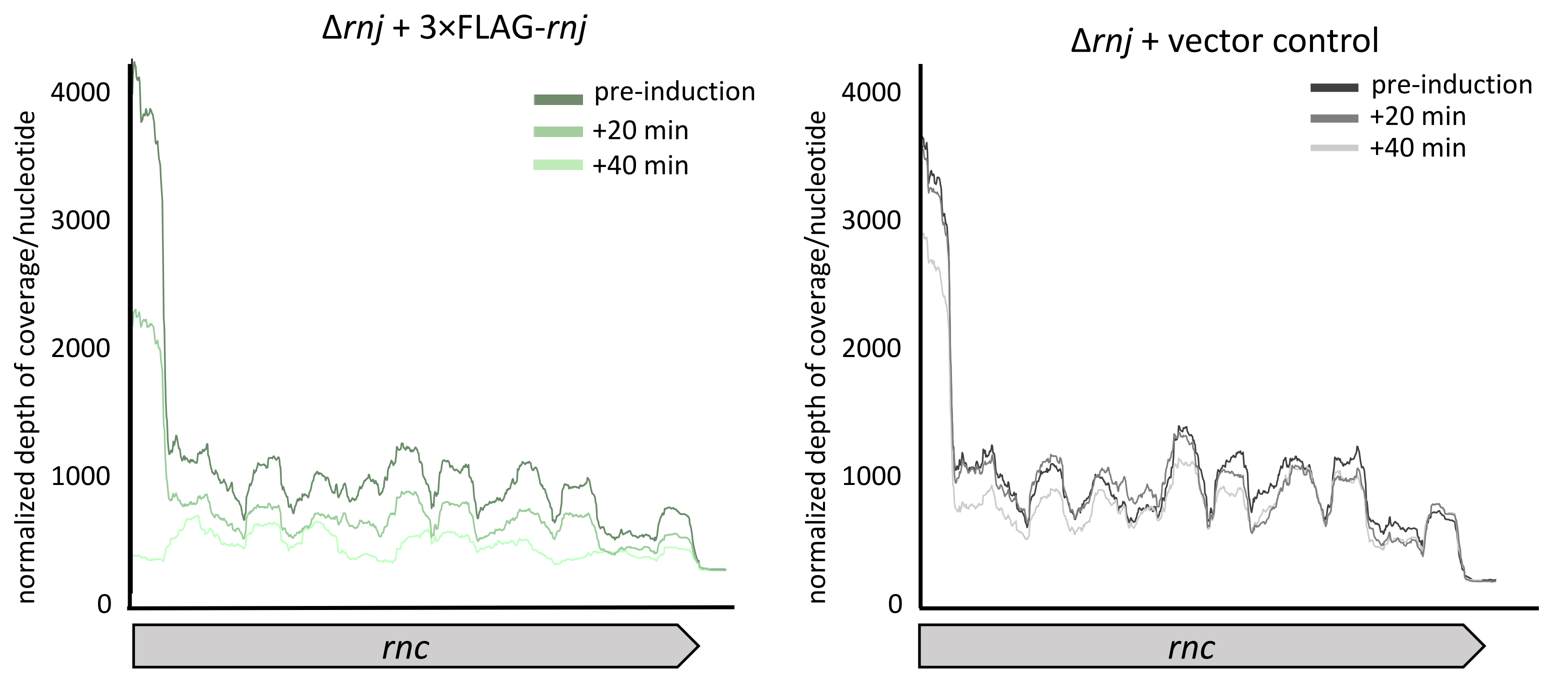

Supplementary Figure 4: *rnc* transcript is directly targeted by RNase J. Second biological replicate for experiment presented in Figure 7B.

Figure S5

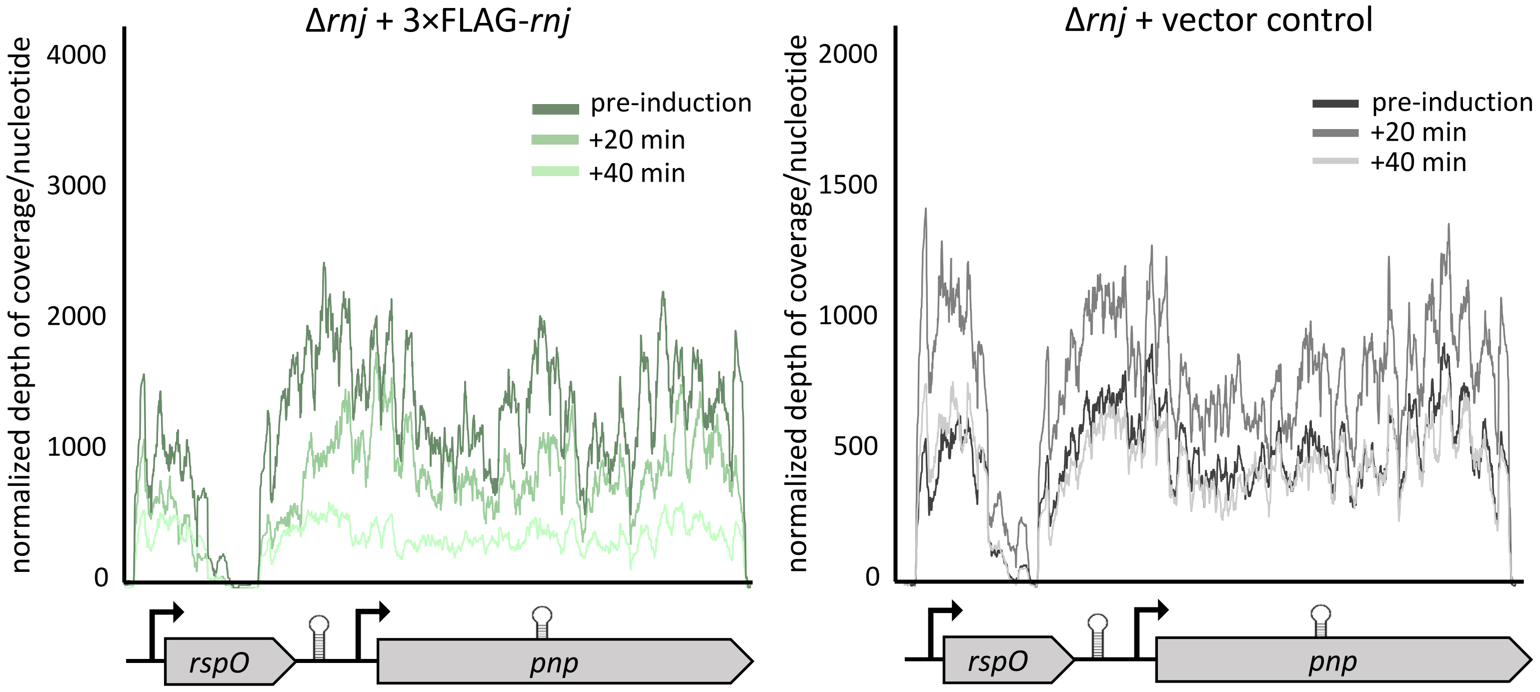

Supplementary Figure 5: *rspO-pnp* transcript is directly targeted by RNase J. Second biological replicate for experiment presented in Figure 7D.
