# Supplementary material for: Defining the networks that connect RNase III and RNase J-mediated regulation of primary and specialized metabolism in Streptomyces venezuelae
Source: J Bacteriol. 2025 Apr 14;207(5):e00024-25. doi: 10.1128/jb.00024-25 (PMC12096830; doi:10.1128/jb.00024-25)
Supplement: Supplemental tables — Tables S6 to S8. [file jb.00024-25-s0004.pdf]

Supplementary Table S6 – Strains used in this study

Strains	Genotype/characteristics/use	Reference
Streptomyces venezuelae NRRL B-65442	Wild type	[1]
E325	*S. venezuelae rnc::aac(3)IV* (*vnz_26040*)	[2]
E326	*S. venezuelae rnj::aac(3)IV* (*vnz_26680*)	[2]
E390	*S. venezuelae chpDFG rdlAB::aac(3)IV* (*vnz_22870-vnz_22985*) *rnc:: hyg* (*vnz_26040*)	This work
E391	*S. venezuelae rsbN::aac(3)IV* (*vnz_15660*)	This work
Streptomyces coelicolor A3(2) M145	Wild type; used to amplify *tipA*	[3]
Escherichia coli DH5α	Routine cloning	Invitrogen
E. coli BW25113/pIJ790	Introducing mutations in cosmid DNA	[4]
E. coli ET12567/pUZ8002	Generation of methylation-free plasmid DNA and conjugation into *Streptomyces*	[4]

Supplementary Table S7 – Plasmids and cosmids used in this study

Cosmid/plasmid	Description	Reference
sv-6-D01	*S. venezuelae* cosmid carrying *rsbN*	Gift from M. Buttner
sv-4-H10	*S. venezuelae* cosmid carrying *rdlAB chpDFG*	Gift from M. Buttner
sv-3-B07	*S. venezuelae* cosmid carrying *rnc*	Gift from M. Buttner
pIJ773	Plasmid carrying the *aac(3)IV-oriT* cassette	[4]
pIJ10700	Plasmid carrying the *hyg-oriT* cassette	[5]
pMS82	Integrative cloning vector: *hyg*, *oriT*, *int* ΦBT1, *attP* ΦBT1	[6]
pMC390	P_{ermE*}-*vnz_19585* (*phoP* D52E) cloned into pMS82	This work
pIJ6902	Integrative cloning vector: *aac(3)IV, tsr*, *oriT*, *int* ϕC31, *attP* ϕC31	[7]
pMC388	pIJ6902::Apra:Hyg::*tipA*; *tipA* sequence from *S. coelicolor* cloned into BglII site of pIJ6902. Apramycin resistance marker replaced with hygromycin resistance marker.	This work
pMC387	pIJ6902::ΔApra:Hyg::riboswitch::*tipA*; theophylline-responsive riboswitch cloned between the *tipA* promoter and *tipA* coding sequence	Riboswitch sequence [8] This work
pMC386	pIJ6902::ΔApra:Hyg::riboswitch::*tipA*::*rnc3×FLAG*; *rnc* sequence with an N-terminal 3×FLAG tag cloned under *tipA* promoter	This work

pMC389	pIJ6902::ΔApra:Hyg::riboswitch::*tipA*::*rnj3×FLAG*; *rnj* sequence with an N-terminal 3×FLAG tag cloned under *tipA* promoter	This work
pGus	Integrative cloning vector: *aac(s)IV*, *aadA*, *oriT*, *int* ϕC31, *attP* ϕC31, *gusA*	[9]
pMC391	Kanamycin resistance gene (*kan*) cloned into pGus	This work
pMC392	*glnA* promoter sequence cloned upstream of promoterless *gusA* in pGus (pMC391)	This work
pMC393	*nirB* promoter sequence cloned upstream of promoterless *gusA* in pGus (pMC391)	This work

Supplementary Table S8 – Oligonucleotides used in this study

Name	Sequence (5' – 3')*	Use
rdlB RED F	GGGCGGCGGGGCCGCAACCGAGCGGAGCGTCTTCGCTCA**ATTCCGGGGATCCGTCGACC**	*chpDFG rdlAB* ReDirect cassette
chpG RED R	TCAGCCGCCGTAGCCGCCGTCACCCTTGTCGTGACCGTCTG**TAGGCTGGAGCTGCTTC**	*chpDFG rdlAB* ReDirect cassette
Rdl int F	CAGGGTTCCCTGAACAAGCC	*chpDFG rdlAB* mutant check
Rdl check up R	TGAAGTGACGGCACACGTA	*chpDFG rdlAB* mutant check
rsbN RED F	GGGAGTCGACCGTCATGACGAGAGGAGGTGCCGCCAGTG**ATTCCGGGGATCCGTCGACC**	*rsbN* ReDirect cassette
rsbN RED R	GGCGCCCCCCGTCCGTGGGGGCGCCCCTTCTTCTTGTCATG**TAGGCTGGAGCTGCTTC**	*rsbN* ReDirect cassette
rsbN up F	CACTCTTTCGTGTGGATGCG	*rsbN* mutant check
rsbN down R	AGCGAGTCACCGGGAAGCG	*rsbN* mutant check
rsbN in R	AGCGAGTCACCGGGAAGCG	*rsbN* mutant check
nirB XbaI F	ATAT<u>TCTAGA</u>GGCGGGAACGGGTGCG	Cloning *nirB* promoter into pGus
nirB KpnI R	ATAT<u>GGTACC</u>ACCGGGAAGCGTGCGC	Cloning *nirB* promoter into pGus
glnA XbaI F	ATAT<u>TCTAGA</u>CCAAGATCCGAGTGCTTGCC	Cloning *glnA* promoter into pGus
glnA KpnI R	ATAT<u>GGTACC</u>CCCACTCCTCCTACTCCC	Cloning *glnA* promoter into pGus
KanR F	CACGCTGCCGCAAGCACTCAGG	Cloning Kan resistance
KanR R	TCAGAAGAACTCGTCAAGAAGGCGA	Cloning Kan resistance
TipA BglII F	ATAT<u>AGATCT</u>CTGACCGAGGTGGTTCCTCC	Amplify *tipA* with BglII restriction sites

TipA BglII R	ATACAGATCTGTCTCACCAAGACGCTGGTCG	Amplify *tipA* with BglII restriction sites
Ribo TipA F	CCAGCATCGTCTTGATGCCCTTGGCAGCACCCTGCTA AGGAGGCAACAAGGTGAGCTACTCCGTGG	Add riboswitch between *tipA* and promoter (overlap extension)
Ribo TipA R	AGGGCATCAAGACGATGCTGGTATCACCGGAACCTAT AGTGAGTCGTACTGACGCCGTCCACGC	Add riboswitch between *tipA* and promoter (overlap extension)
PhoP F	CATCATCATATGGTGACCCGAGTGCTTGT	Cloning *phoP* into pMS82
PhoP R	CATCATCTCGAGGGAACACATGAAGGGGC	Cloning *phoP* into pMS82
PhoP D52E F	CTCCTCGAGCTGATGC	Site directed mutagenesis of *phoP* (D52E; overlap extension)
PhoP D52E R	GCATCAGCTCGAGGAG	Site directed mutagenesis of *phoP* (D52E; overlap extension)
3xF NdeI F	ATATCATATGGACTACAAGGACCACGACGG	Cloning either *rnc* or *rnj* 3xFLAG (binds FLAG sequence) into pIJ6902
rnj R	CATCATGGTACCCCCGTTCTGGCGGAGC	Cloning *rnj* into pIJ6902
rnc R	ATATGGTACCGACAGCGACTCAACC	Cloning *rnc* into pIJ6902

*Bold: resistance cassette-specific sequences; underlined: engineered restriction enzyme sites

References

[1] Gomez-Escribano JP, Holmes NA, Schlimpert S, Bibb MJ, Chandra G, Wilkinson B, Buttner MJ, Bibb MJ. 2021. *Streptomyces venezuelae* NRRL B-65442: genome sequence of a model strain used to study morphological differentiation in filamentous actinobacteria. J Ind Microbiol Biotechnol. 1(48):9–10.

[2] Jones SE, Leong V, Ortega J, Elliot MA. 2014. development, antibiotic production, and ribosome assembly in *Streptomyces venezuelae* are impacted by RNase J and RNase III deletion. J Bacteriol. 196(24):4253–4267.

[3] Keiser T, Bibb M, Buttner M, Chater K. 2000. Practical *Streptomyces* Genetics.

[4] Gust B, Challis GL, Fowler K, Kieser T, Chater KF. 2003. PCR-targeted *Streptomyces* gene replacement identifies a protein domain needed for biosynthesis of the sesquiterpene soil odor geosmin. Proc Natl Acad Sci. 100(4):1541–1546.

[5] Gust B, Chandra G, Jakimowicz D, Yuqing T, Bruton CJ, Chater KF. 2004. λ *red*-mediated genetic manipulation of antibiotic-producing *Streptomyces*. Adv Appl Microbiol. 54:107–128.

[6] Gregory MA, Till R, Smith MCM. 2003. Integration site for *Streptomyces* phage ϕBT1 and development of site-specific integrating vectors. J Bacteriol. 185(17):5320–5323.

[7] Huang J, Shi J, Molle V, Sohlberg B, Weaver D, Bibb MJ, Karoonuthaisiri N, Lih C, Kao CM, Buttner MJ, Cohen SN. 2005. Cross-regulation among disparate antibiotic biosynthetic pathways of *Streptomyces coelicolor*. Mol Microbiol. 58(5):1276–1287.

[8] Kamiura R, Toya Y, Matsuda F, Shimizu H. 2019. Theophylline-inducible riboswitch accurately regulates protein expression at low level in *Escherichia coli*. Biotechnol Lett. 41(6–7):743–751.

[9] Myronovskyi M, Welle E, Fedorenko V, Luzhetskyy A. 2011. β-Glucuronidase as a sensitive and versatile reporter in actinomycetes. Appl Environ Microbiol. 77(15):5370–5383.
